# A European Spectrum of Pharmacogenomic Biomarkers: Implications for Clinical Pharmacogenomics

**DOI:** 10.1371/journal.pone.0162866

**Published:** 2016-09-16

**Authors:** Clint Mizzi, Eleni Dalabira, Judit Kumuthini, Nduna Dzimiri, Istvan Balogh, Nazli Başak, Ruwen Böhm, Joseph Borg, Paola Borgiani, Nada Bozina, Henrike Bruckmueller, Beata Burzynska, Angel Carracedo, Ingolf Cascorbi, Constantinos Deltas, Vita Dolzan, Anthony Fenech, Godfrey Grech, Vytautas Kasiulevicius, Ľudevít Kádaši, Vaidutis Kučinskas, Elza Khusnutdinova, Yiannis L. Loukas, Milan Macek, Halyna Makukh, Ron Mathijssen, Konstantinos Mitropoulos, Christina Mitropoulou, Giuseppe Novelli, Ioanna Papantoni, Sonja Pavlovic, Giuseppe Saglio, Jadranka Setric, Maja Stojiljkovic, Andrew P. Stubbs, Alessio Squassina, Maria Torres, Marek Turnovec, Ron H. van Schaik, Konstantinos Voskarides, Salma M. Wakil, Anneke Werk, Maria del Zompo, Branka Zukic, Theodora Katsila, Ming Ta Michael Lee, Alison Motsinger-Rief, Howard L. Mc Leod, Peter J. van der Spek, George P. Patrinos

**Affiliations:** 1 Erasmus University Medical Center, Faculty of Medicine, Department of Bioinformatics, Rotterdam, the Netherlands; 2 University of Malta, Faculty of Medicine and Surgery, Department of Physiology and Biochemistry, Msida, Malta; 3 University of Patras School of Health Sciences, Department of Pharmacy, Patras, Greece; 4 Center for Proteomic and Genomic Research, Observatory, Cape Town, South Africa; 5 King Faisal Specialist Hospital and Research Centre, Riyadh, Saudi Arabia; 6 University of Debrecen, Debrecen, Hungary; 7 Boğaziçi University, Istanbul, Turkey; 8 University of Kiel, Institute for Experimental and Clinical Pharmacology, Kiel, Germany; 9 University of Malta, Department of Applied Biomedical Science, Faculty of Health Sciences, Msida, Malta; 10 University of Rome “Tor Vergata”, Department of Biomedicine and Prevention, Rome, Italy; 11 University Hospital Centre, Zagreb, Croatia; 12 Institute of Biochemistry and Biophysics, Polish Academy of Sciences, Warsaw, Poland; 13 University of Santiago de Compostela, Santiago, Spain; 14 University of Cyprus, Molecular Medicine Research Center, Department of Biological Sciences, Nicosia, Cyprus; 15 University of Ljubljana Faculty of Medicine, Ljubljana, Slovenia; 16 University of Malta, Faculty of Medicine, Department of Surgery, Msida, Malta; 17 Department of Human and Medical Genetics, Faculty of Medicine, Vilnius University, Vilnius, Lithuania; 18 Comenius University, Faculty of Natural Sciences, Bratislava, Slovakia; 19 Center for Molecular Medicine, Slovak Academy of Sciences, Bratislava, Slovakia; 20 Institute of Biochemistry and Genetics, Ufa Scientific Center, Russian Academy of Sciences, Ufa, Russia; 21 Department of Genetics and Fundamental Medicine, Bashkir State University, Ufa, Russia; 22 University of Athens, Faculty of Pharmacy, Department of Pharmaceutical Chemistry, Athens, Greece; 23 Charles University, 2nd Faculty of Medicine and University Hospital Motol, Prague, Czech Republic; 24 Institute of Hereditary Pathology, Ukrainian National Academy of Medical Sciences, Lviv, Ukraine; 25 Erasmus University Medical Center, Department of Clinical Chemistry, Rotterdam, the Netherlands; 26 The Golden Helix Foundation, London, United Kingdom; 27 Institute of Molecular Genetics and Genetic Engineering University of Belgrade, Laboratory of Molecular Biomedicine, Belgrade, Serbia; 28 University of Turin School of Medicine, Turin, Italy; 29 University of Zagreb School of Medicine, Zagreb, Croatia; 30 University of Cagliari, Department of Biomedical Sciences, Cagliari, Italy; 31 RIKEN Institute, Center for Genomic Medicine, Laboratory for International Alliance, Yokohama, Japan; 32 North Carolina State University, Department of Statistics, Raleigh, NC, United States of America; 33 Moffitt Cancer Center, Tampa, FL, United States of America; Universite de Montreal, CANADA

## Abstract

Pharmacogenomics aims to correlate inter-individual differences of drug efficacy and/or toxicity with the underlying genetic composition, particularly in genes encoding for protein factors and enzymes involved in drug metabolism and transport. In several European populations, particularly in countries with lower income, information related to the prevalence of pharmacogenomic biomarkers is incomplete or lacking. Here, we have implemented the microattribution approach to assess the pharmacogenomic biomarkers allelic spectrum in 18 European populations, mostly from developing European countries, by analyzing 1,931 pharmacogenomics biomarkers in 231 genes. Our data show significant inter-population pharmacogenomic biomarker allele frequency differences, particularly in 7 clinically actionable pharmacogenomic biomarkers in 7 European populations, affecting drug efficacy and/or toxicity of 51 medication treatment modalities. These data also reflect on the differences observed in the prevalence of high-risk genotypes in these populations, as far as common markers in the *CYP2C9*, *CYP2C19*, *CYP3A5*, *VKORC1*, *SLCO1B1* and *TPMT* pharmacogenes are concerned. Also, our data demonstrate notable differences in predicted genotype-based warfarin dosing among these populations. Our findings can be exploited not only to develop guidelines for medical prioritization, but most importantly to facilitate integration of pharmacogenomics and to support pre-emptive pharmacogenomic testing. This may subsequently contribute towards significant cost-savings in the overall healthcare expenditure in the participating countries, where pharmacogenomics implementation proves to be cost-effective.

## Introduction

Pharmacogenomics (PGx) aims to delineate individual differences in drug use, both in terms of efficacy and toxicity, with the underlying genetic profile [1]. There is growing evidence suggesting that variants in genes encoding for drug metabolizing enzymes and transporters directly impact their function, which is translated in both adverse drug reactions (ADRs) and/or altered efficacy for a number of relevant drugs. As such, most medications will be beneficial to almost half of the treated patients, while the remaining half will either go untreated or, worse, develop mild-to-serious ADRs, which constitute one of the leading causes of mortality and morbidity [2]. From a plethora of data in the scientific literature, correlating genomic variants with drug response, the main regulatory bodies, namely the United States Food and Drug Administration (FDA; www.fda.gov) and the European Medicines Agency (EMA; www.ema.europa.eu) have shortlisted more than 120 drugs where there is strong scientific evidence to support the influence of genomic biomarkers over dosing, safety risk, or efficacy [3–5].

There are also significant geographic differences in the prevalence of several ADRs and medication efficacy [6,7]. This is not unexpected considering the fact that these differences in drug response are also correlated with genomic variants, whose allelic frequencies vary among different population and racial groups. Interestingly, there are distinct genetic differences within European populations as evidenced through investigations on the distribution of paternal traits located on the Y-chromosome [8]. As with every genomic variant, PGx biomarkers that have been associated with both adverse events and variable drug efficacy also have significant geographic variability, as far as their allelic frequencies are concerned.

So far, population genomics studies have almost exclusively focused on genomic variants dispersed across the genome, with very little emphasis being given to the global prevalence and relevance of PGx biomarkers. To this end, there is very limited information related to the variable allele frequency of PGx biomarkers in different population, rather than racial, groups.

Here, we have implemented the microattribution approach [9,10] to comprehensively sketch a pan-European PGx biomarkers spectrum, by analyzing 1,931 clinically relevant PGx biomarkers in 231 genes in 11 European populations, namely Croatian, Czech, Dutch, German, Greek, Hungarian, Maltese, Polish, Serbian, Slovenian and Turkish and followed up for 36 actionable PGx biomarkers in 7 additional European populations, namely Cypriot, Italian, Lithuanian, Russian, Slovakian, Spanish and Ukrainian. We report important population-specific differences in the prevalence of clinically actionable pharmacogenes, which are reflected in rationalizing drug dose for several of the most commonly prescribed drugs, for which PGx information is available in their labels. These data provide the basis for being replicated in larger population samples in these countries, which can then be exploited not only to develop guidelines for medical prioritization, but most importantly also to facilitate integration of PGx in these countries.

## Methods

### Sample collection using the microattribution approach

A total of 1,710 subjects, all of them healthy volunteers, were analyzed for this study. In all, 1,105 subjects were analyzed (847 with the high-throughput Affymetrix DMET^™^ Plus platform and 258 with conventional genotyping; see below) from a total of 18 European countries and compared against 499 subjects from the Middle East (Saudi Arabia) and 106 subjects from South Africa (Table 1). For every population sample, we included subjects that were representative of the population of the country in question, by ensuring that their parents were also natives and not immigrants, especially in those countries with high immigration rates. Also, we have compared our European populations against Saudi Arabian and South African populations since they differ both geographically and genetically.

**Table 1:** Sample composition.

Population	Number of samples	
	Affymetrix DMET^™^ Plus platform analysis	Follow-up genotyping
Croatian	45	-
Cypriot	-	40
Czech	42	-
Dutch	349	-
German	97	-
Greek	44	-
Hungarian	48	-
Italian	-	29
Lithuanian	-	20
Maltese	41	-
Polish	46	-
Russian	-	39
Serbian	46	-
Slovak	-	26
Slovenian	48	-
Spanish	-	52
Turkish	41	-
Ukrainian	-	52
**SUBTOTAL—European**	**847**	**258**
Saudi Arabian	499	-
**SUBTOTAL—Middle East**	**499**	-
South African (Caucasian)	35	-
South African (Mixed)	36	-
South African (Xhosa)	35	-
**SUBTOTAL—South African**	**106**	-
**TOTAL**	**1.452**	**258**

The recruitment of subjects and DNA extraction from peripheral blood or saliva were performed locally in the various participating centers in each country, ensuring that written informed consent was obtained following approval by local and/or regional ethical bodies. Research described in this study has been approved by the Institutional Review Board (IRB) of all participating Institutes: Cyprus National Bioethics Committee (Cyprus), Ethics Committee of the Medical Faculty, University of Kiel (Germany), Ethics Committee of the Teaching Hospital of Cagliari, Ethics Committee of Policlinico "Tor Vergata”, Rome and Ethics Committee of the San Luigi University Hospital, Orbassano-Torino (Italy), Regional Ethics Committee of the University of Debrecen (Hungary), Ethics committee of the Institute of Biochemistry and Genetics, Ufa Scientific Center of Russian Academy of Sciences (Russian Republic), Ethics Committee of University Children’s Hospital Belgrade (Serbia), Ethics Committee of the Erasmus MC Hospital, Rotterdam (the Netherlands), Ethics Committee of Bogazici University for Human Research (INAREK), Bogazici (Turkey), Ethics Committee of the King Faisal Specialist Hospital and Research Centre, Riyadh (Saudi Arabia), Republic of Slovenia National Medical Ethics Committee, Ljubljana (Slovenia), Internal Ethics Committee of University Hospital Motol, Prague (Czech Republic), Republic of Lithuania Vilnius Regional Medical Ethics Committee, Vilnius (Lithuania), Ethics Committee of the University of Patras, Patras (Greece), The University Research Ethics Committee (UREC) of the University of Malta, Msida (Malta), Ethics Committee of the Medical University of Warsaw, Warsaw (Poland), Ethics Committee of Slovak Academy of Sciences, Bratislava (Slovakia), Institute of Hereditary Pathology of Ukrainian National Academy of Medical Sciences, Lviv (Ukraine), Ethics Committee of the Zagreb University, Hospital Center, Zagreb (Croatia), Human Research Ethics Committee of the UCT Faculty of Health Sciences, Cape Town (South Africa).

### Genotyping methods

De-identified DNA samples, contributed with minimal demographic information (country of origin and gender) were genotyped by the Affymetrix DMET^™^ Plus platform (Affymetrix Inc., Santa Clara, CA, USA), according to the manufacturer’s instructions. The full list of SNPs and associated genes that were analyzed are available and can be accessed from http://www.affymetrix.com/catalog/131412/AFFY/DMET-Plus-Solution#1_3. Low-quality data were excluded from subsequent analysis, based on genotyping efficiency, both in terms of variant calls [variant excluded if absent (no-call) from >5% of the individuals] and samples (sample excluded if more than 5% of variants were missing) [11]. All resulting Affymetrix .cel and .arr files were analyzed using the Affymetrix DMET^™^ Console software. In particular, we calculated the aggregated PGx biomarker allele frequencies in all 11 populations analyzed by the Affymetrix DMET^™^ Plus platform (Table A in S1 File), as well as the prevalence of the individual haplotypes showing the metabolizer status of the population sample included in our analysis (Table B in S1 File).

For validation purposes, 36 actionable PGx biomarkers were selected, based on their clinical utility, and genotyped using TaqMan^®^ SNP Genotyping Assays (Thermo Fisher Scientific, Waltham, MA, USA) and/or Sanger sequencing. The reagent volumes and RT-PCR conditions were according to manufacturer’s instructions. The full list of SNPs and their TaqMan^®^ Assay ID’s are provided in Table C in S1 File. Raw data from genotyping experiments were analyzed using the TaqMan^®^ Genotyper software and the aggregated PGx biomarker allele frequencies are provided in Table A in S1 File.

### Data deposition and sharing

Genotype data from the samples contributed by each participating center was stored in a de-identified aggregated level in FINDbase database for clinically relevant genomic variation allele frequencies [12], based on the microattribution approach [9,10], using the unique ResearcherID (www.reseacherid.com) of all participating investigators.

### Principal Components Analysis (PCA)

To calculate eigen values and eigen vectors for the first principal components, we removed the only triallelic SNP to pass quality control. The allele frequencies for 1,804 SNPs were analyzed via Past 3 software package. http://folk.uio.no/ohammer/past/

### Hierarchal Clustering

To build a hierarchy cluster using Euclidean Distance as a metric we used the nearest neighbor and classical algorithms via the software package Past3. The allele frequencies of 1,804 SNPs for each ethnicity were used in these statistical analyses.

### STRUCTURE Analysis

Structure analysis was conducted through STRUCTURE version 2.3.4. The individual genotype was used for this analysis. European genotypes were compared to the Saudi Arabians and South Africans. The burn in period and number of MCMC reps were both set to 1000. The ancestry and frequency modeling parameters were kept at their default settings. Finally, we set the number of populations (K) to 3, purporting three main global clusters.

### Warfarin dose prediction

Averages for the predicted warfarin dose for each country were calculated using the published International Warfarin Pharmacogenomics Consortium (IWPC) algorithm [13]. Because individual information for height, weight and age was not known, these values were approximated and set equal for each individual to the Caucasian racial group average [14]. These values were used along with individual genotypes for *CYP2C9* and *VKORC1* genes to calculate dosing, and averages were made across individuals in each country.

### Determination of actionable genotypes

Lastly, we analyzed the occurrence of actionable genotypes among our study groups in all European population cumulatively and compared against the Saudi Arabian and South African populations. By “actionable”, we refer to those genotypes that lead to genotype-guided advice for a change in dose or medication overall, while by “high risk”, we refer to those genotypes that may lead to a severe adverse drug reaction. Such genotypes are: (i) homozygous CYP2C19*2 (rs424485), leading to clopidogrel resistance, (ii) homozygous SLCO1B1*5 (rs4149056), leading to simvastatin-induced myopathy, (iii) homozygous CYP2C9*3 (rs1057910), leading to warfarin sensitivity, and (iv) homozygous or compound heterozygous TPMT*2 (rs1800462) or TPMT*3 (rs1800460, rs1142345), associated with thiopurine-induced myelotoxicity. As such, an individual who is found to be homozygous for the SLCO1B1*5 (rs4149056) variant is at a much higher (20-fold, compared to homozygous normal) risk of developing simvastatin-induced myopathy, compared to SLCO1B1*5 (rs4149056) heterozygotes (4-fold risk, compared to homozygous normal) or homozygous normal individuals [15,16]. A list of all actionable and high-risk genotypes was obtained from the Vanderbilt PREDICT program [17].

## Results

Our genotyping effort consisted of two phases. Phase I included the analysis of 1,931 PGx variants in 231 pharmacogenes, using the Affymetrix DMET^™^ Plus platform, for 847 samples from 11 European populations, which were subsequently compared against 499 samples from the Saudi Arabian population and 106 samples from South African populations (Table 1). Phase II consisted of the validation in 258 samples from 7 additional European populations and focused on 36 clinically actionable PGx biomarkers in 9 pharmacogenes; *CYP2C19*, *CYP2C9*, *CYP2D6*, *VKORC1*, *DPYD*, *UGT1A1*, *TPMT*, *NAT2*, and *SLC01B1*.

### Prevalence of PGx biomarkers in European populations

Initially, we explored the genotyping results obtained from the analysis of 847 individuals from 11 European populations, using the Affymetrix DMET^™^ Plus platform (Table 1).

**Fig 1:**
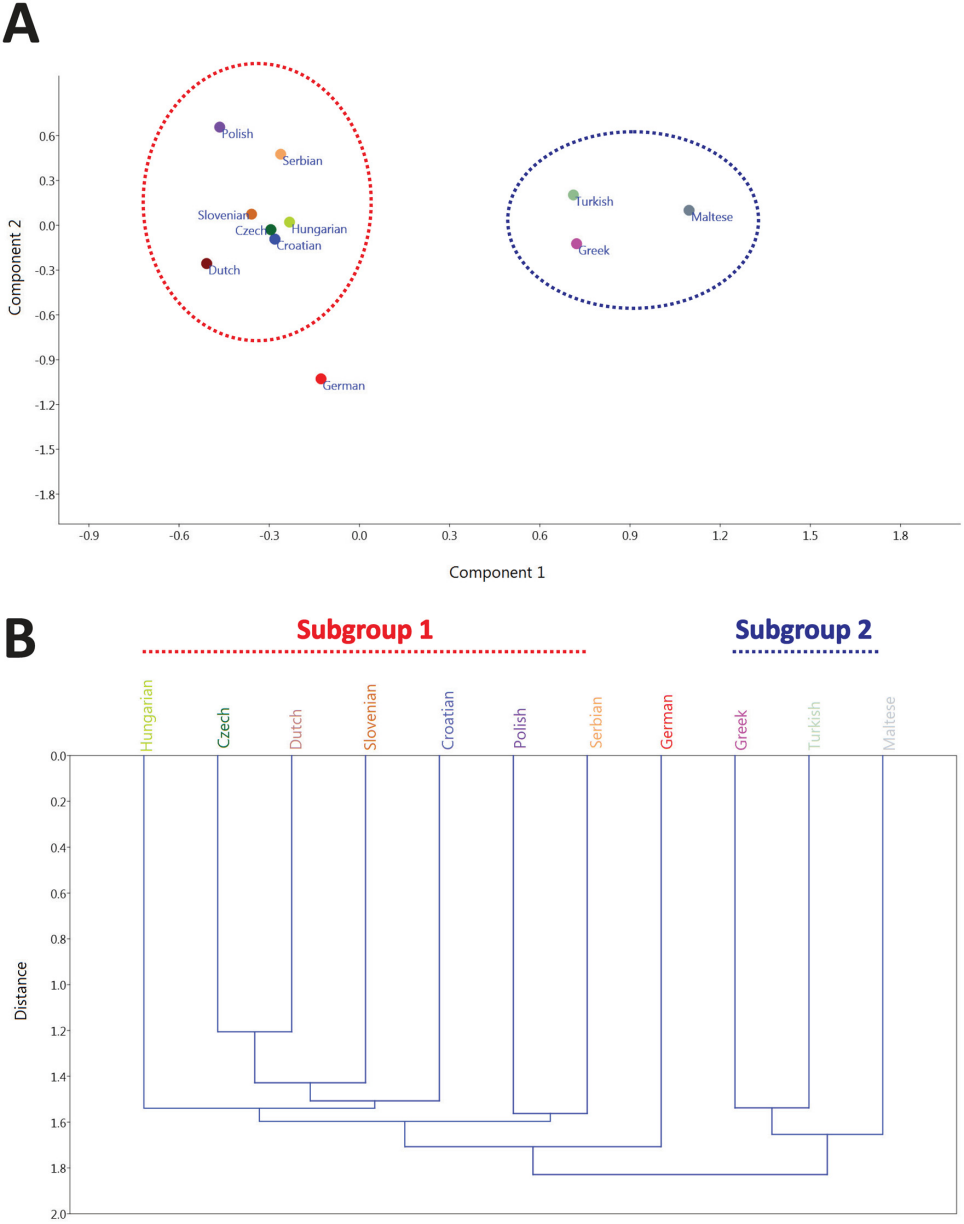
A. Principal Components Analysis, using allele frequencies calculated for each population, analyzed via Past 3 software package. http://folk.uio.no/ohammer/past/. B. Hierarchical clustering using Euclidean Distance as a metric, using the Past3 software package, based on the allele frequencies for each population (see also Methods for details). The two distinct population subgroups are indicated with different colors.

Subsequently, we have performed principal component analysis to compare the PGx marker allele frequencies for all variants identified in the 11 European populations as well as against 499 and 106 individuals from Saudi Arabian and South African descent, respectively (Table 1). Our analysis indicated differences, not only among the South African, Saudi Arabian and European populations, as one would expect, but also among European populations. In particular, the German population appears to cluster further away from the rest of the European populations, while there are two other subgroups among European populations, namely Czech, Croatian, Dutch, Hungarian, Polish, Serbian, and Slovenian (subgroup 1) and Greek, Maltese and Turkish (subgroup 2; Fig 1A). Of course, Germany clusters well within the European Caucasian populations and away from the Saudi Arabian and South African populations tested (data not shown). Notably, even populations thought to be more divergent, such as the Greek or Turkish and the Maltese, appear to cluster in the same subgroup. The same pattern was also obtained, using hierarchical clustering (Fig 1B), indicating that the European sample analyzed can be indeed clustered in two distinct subgroups. The PGx variant allele frequencies for all populations analyzed have been deposited into FINDbase, a clinically relevant genomic variant allele frequency database, using the microattribution approach.

Next, we attempted to assess the overall diversity within each population by estimating the percentage of the Affymetrix DMET^™^ Plus platform variants derived from three major HapMap ancestral populations, namely Caucasians (CEU), Yoruba (YRI) and Asians (from the Japanese and Chinese samples, JPT and CHB respectively) [14], using the STRUCTURE algorithm [18]. Our data showed that the contribution of reference genomes generally followed the geographic origin of the population, and showed no significant differences among the European populations analyzed (Figure A in S2 File).

### Replication of findings in actionable PGx markers in European populations

Subsequently, we have focused our analysis on 36 clinically actionable PGx biomarkers in 9 pharmacogenes (*CYP2D6*, *CYP2C19*, *CYP2C9*, *DPYD*, *TPMT*, *NAT2*, *SLCO1B1*, *VKORC1*, and *UGT1A1*), namely PGx variants that have been approved by regulatory agencies (US Food and Drug Administration (www.fda.gov) and/or European Medicines Agency (www.ema.europa.eu) to be used for clinical PGx. Our data from analyzing the prevalence of those 36 PGx variants in all 18 European populations (Table A in S1 File) indicate that there are significant differences in the prevalence of 7 PGx biomarkers in at least 7 European populations, which are implicated in 51 drugs’ efficacy and toxicity (Table 2). In particular, the prevalence of the rs4149056 (SLCO1B1*5) variant appears to be significantly different in 3 European populations, namely Polish, Cypriot and Lithuanian, compared to the European average. Similarly, the prevalence of the rs12248560 (CYP2C19*17) appears to be statistically different in the Greek and Polish populations, the rs1057910 (CYP2C9*3), one of the key markers to rationalize warfarin treatment, seems to be statistically different in the Dutch and Polish populations, the rs1135840 (CYP2D6*2/*2XN/*39), rs3892097 (CYP2D6*4/*4F/*4G/*4H), and rs1065852 (CYP2D6*10) markers, involved in the metabolism of over 30 antipsychotic, antidepressant and other drugs, appears to be statistically different in the Slovak, Polish and Spanish populations, respectively, and the rs1801280 (NAT2*4/*5A/*5B/*5C/*5D/*5E/*5G/*5J/*14C/*14F) is different in the Greek population (Table 2). These findings can help towards rationalizing treatment modalities for these drugs in the respective countries and possibly amend existing treatment recommendations.

**Table 2:** Outline of the significant differences (p-values<0.05 in boldface) of the prevalence of actionable PGx biomarkers in European populations, compared to the average European.

Gene	PGx variant	Population							Relevant drugs
	rs number ^a^	Dutch	Greek	Polish	Cypriot	Lithuanian	Slovak	Spanish	
***CYP2C19***	rs12248560 (*17)	0.862	**0.011**	**0.042**	0.056 ^b^	0.622	0.113	0.862	Citalopram, Clopidogrel, Omeprazole
***CYP2C9***	rs1057910 (*3)	**0.024**	1.000	**0.035**	1.000	0.806	0.374	0.631	Warfarin
***CYP2D6***	rs1135840 (*2/*2XN/*39)	0.887	0.316	0.479	0.196	0.673	**0.042**	0.887	Amitriptyline, Carvedilol, Codeine, Metoprolol, Nortriptyline, Tamoxifen, Tramadol, Trimipramine, Venlafaxine
	rs3892097 (*4)	0.716	1.000	**0.003**	0.478	0.293	0.089	**0.004**	
	rs1065852 (*10)	0.859	0.603	**0.004**	0.859	0.309	0.099	0.459	
***NAT2***	rs1801280 (*4/*5A/*5B/*5C/*5D/*5E/*5G/*5J/*14C/*14F)	0.322	**0.039**	0.777	0.247	1.000	0.670	0.667	Hydralazine, Isoniazid,
***SLCO1B1***	rs4149056 (*5)	0.69647	0.308	**0.006**	**0.048**	**0.001**	0.476	1.000	Simvastatin

^a^: Star allele nomenclature, where applicable

^b^: Statistical trend

**Fig 2:**
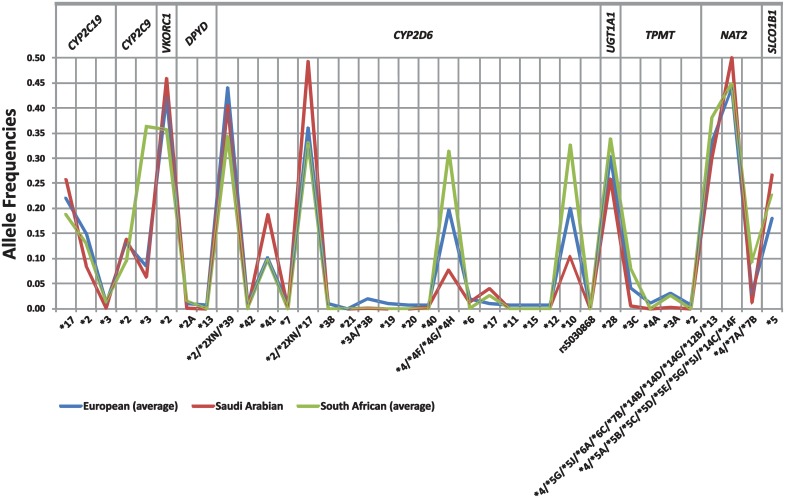
Comparison of the frequencies (vertical axis; %) of the 36 actionable PGx biomarkers (depicted at the horizontal axis) among European, Saudi Arabian and South African populations.

Subsequently, the frequencies of those 36 actionable PGx biomarkers have been assessed cumulatively for all European population analyzed and compared against those in the Saudi Arabian and South African populations (Fig 2). Our data indicate that there are some distinct differences among these populations, with direct impact on drug use in these populations. In particular, the prevalence of the rs1057910 (CYP2C9*3), that is correlated with warfarin treatment efficacy is significantly higher in the South African population (0.36) compared to the European populations analyzed (0.08) and the Saudi Arabian population (0.06). The same is true for the rs3892097 (CYP2D6*4/*4F/*4G/*4H) and rs1065852 (CYP2D6*10) PGx variants, related with the metabolism of various psychotropic drugs (0.31–0.33 for the South African population vs 0.20–0.20 for the European populations and 0.08–0.10 for the Saudi Arabian population, respectively), the rs1142345 (TPMT*3C) PGx variant, responsible for thiopurines toxicity (0.08 South African population vs 0.04 for the European populations and 0.01 for the Saudi Arabian population, respectively) and the rs1799931 (NAT2*4/*7A/*7B) PGx variant (0.09 South African population vs 0.03 for the European populations and 0.01 for the Saudi Arabian population, respectively), related to the anti-tuberculosis treatment. On the contrary, the prevalence of the rs1135840 (CYP2D6*2/*2XN/*39) PGx variant was found to be higher in the European populations (0.44), compared to the Saudi Arabian (0.41) and the South African populations (0.34), respectively, while the rs16947 (CYP2D6*2/*2XN/*17) PGx variant seems to be significantly more frequent in the Saudi Arabian population (0.49), compared to the European and South African populations that is present in almost comparable frequencies (0.36 and 0.33, respectively). The latter 2 PGx variants are responsible for the metabolism of antipsychotic and antidepressant drugs.

**Fig 3:**
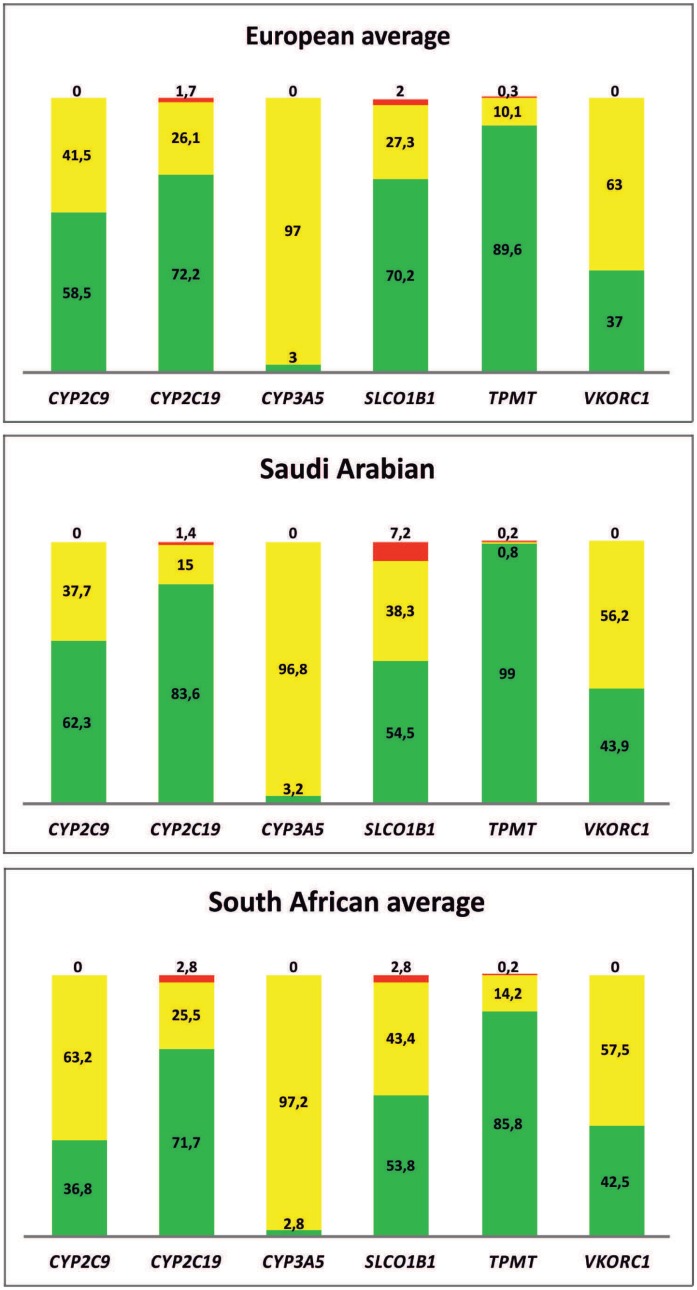
Frequency of the clinically actionable genotypes in the European patients analyzed using the Affymetrix DMET^™^ Plus platform. Green depicts genotypes with no actionable pharmacogenomic biomarkers, yellow depicts genotypes with at least one actionable pharmacogenomic biomarker, and red depicts genotypes with at least one high-risk actionable pharmacogenomic biomarker. As stated in PharmGKB, the term “actionable” does not discuss genetic or other testing for gene/protein/chromosomal variants, but does contain information about changes in efficacy, dosage or toxicity due to such variants.

Furthermore, we have attempted to demonstrate genotype differences among actionable PGx alleles, particularly focusing on the prevalence of high-risk alleles. Specifically, we have narrowed down the list of actionable pharmacogenes and assessed the prevalence of actionable low- and high-risk genotypes from the Vanderbilt PREDICT program [17; see also Methods section]. Our data indicate that the risk of bearing a high-risk genotype in the *CYP2C9*, *CYP2C19*, *CYP3A5*, *VKORC1*, *SLCO1B1*, and *TPMT* varies significantly among European, Saudi Arabian and South African populations (Fig 3). Also, the prevalence of homozygotes for the SLCO1B1*5 (rs4149056) allele is significantly higher in the Saudi Arabian population (7.2%), compared to the South African (2.7%) and the European populations (2%), respectively. Also, the prevalence of the CYP2C9*2 (rs1799853) and CYP2C9*3 (rs1057910) alleles heterozygotes, related to warfarin treatment, is significantly higher in the South African population (63.2%), compared to the Saudi Arabian (37.7%) and European populations (41.5%; Fig 3), indicating a potential higher risk for bleeding based on standard dosages and thus and increased benefit in the South African population). When comparing European populations individually, the Slovenian and Czech populations completely lack individuals with high-risk genotypes for all 6 genes analyzed, namely *CYP2C9*, *CYP2C19*, *CYP3A5*, *VKORC1*, *SLCO1B1*, and *TPMT*, while there are some marked differences among populations related to high-risk *SLCO1B1* genotypes related to simvastatin-induced myopathy and *CYP2C19* genotypes related to inefficient activation of the platelet inhibitor clopidogrel [highest for the Polish population (7%) and the Greek population (4.5%), respectively; not shown].

However, the small number of samples analyzed does not allow for proper statistical analysis of the findings.

### Focus on correlation between CYP2C9 and VKORC1 variants and warfarin dose

PGx contributes towards minimizing adverse drug reactions in a variety of treatment modalities, including e.g. malaria, tuberculosis, pain control, HIV, or major depression [reviewed in 1]. As such, we have attempted to assess whether the observed differences in the prevalence of PGx biomarkers can reflect on the estimated drug dose across all populations analyzed. The vitamin k-antagonist warfarin is the most commonly used drug in anticoagulation therapy, but is characterized by a broad inter-individual variability in dose requirements and a narrow therapeutic index [19]. Warfarin is the only medication for which a genome-guided predictive dosing model is available from the International Warfarin Pharmacogenomics Consortium (IWPC), based on both demographic and genetic (*CYP2C9*, *VKORC1*) factors [13], allowing individual estimation of the warfarin starting dose.

**Fig 4:**
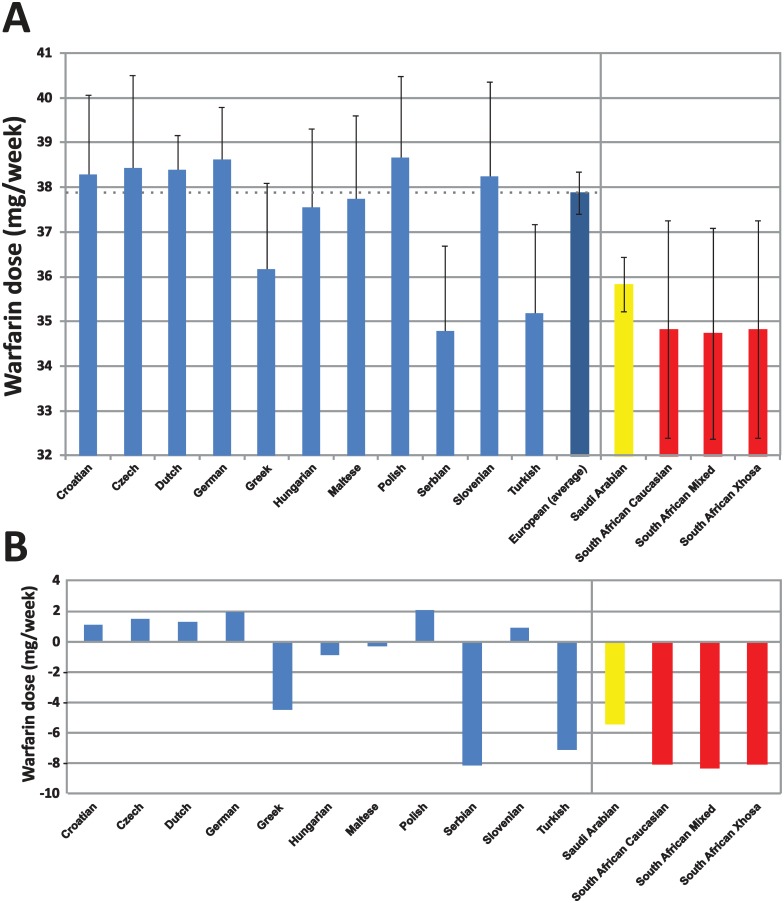
A. Average predicted warfarin dose across individuals for each population. Values for height, weight and age were approximated and set equal as the average of Caucasian racial group and subsequently used along with individual genotypes for *CYP2C9* and *VKORC1* pharmacogenes. B. Average predicted warfarin dose across individuals for each population corrected against the average European dose. Predicted doses were simulated using IWPC.

Based on phenotype prediction (not taking into account patient demographics, such as age, weight, etc), our data indicate that there is an average higher simulated dose requirement for European populations (37.8 mg/week; average dose), which is significantly different compared to the Saudi Arabian and South African populations [35.8 and 34.8 mg/week, respectively (p<0.0001 and p = 0.01, respectively); Table 3]. Furthermore, among the 11 populations analyzed, the Turkish and Serbian populations show a statistically significant difference in the predicted weekly warfarin dose, which is lower, compared to the average European predicted average weekly warfarin dose [35.2 (p = 0.03) and 34.8 (p = 0.004) respectively; Fig 4].

**Table 3:** Outline of the predicted average warfarin dosage calculation for all populations. This table suggests the weekly average dosage along with the standard deviation, confidence interval (95%) and the respective upper bound and lower bound for each population.

Population	Average	St-Dev	Confidence interval 95%	Upper bound	Lower bound
Croatian	38.29	5.85	1.77	40.06	36.52
Czech	38.44	6.57	2.06	40.50	36.38
Dutch	38.38	7.43	0.78	39.16	37.60
German	38.62	5.91	1.18	39.80	37.45
Greek	36.17	6.27	1.92	38.09	34.25
Hungarian	37.55	6.04	1.77	39.31	35.78
Maltese	37.75	6.07	1.86	39.61	35.89
Polish	38.66	6.09	1.82	40.48	36.84
Serbian	34.79	6.62	1.91	36.70	32.87
Slovenian	38.24	7.29	2.13	40.37	36.11
Turkish	35.17	6.27	1.99	37.17	33.18
European (average)	37.88	6.96	0.47	38.36	37.41
Saudi Arabian	35.83	6.92	0.61	36.44	35.23
South African Caucasian	34.83	7.32	2.43	37.26	32.41
South African Mixed	34.74	7.24	2.36	37.10	32.37
South African Xhosa	34.83	7.32	2.43	37.26	32.41

**Fig 5:**
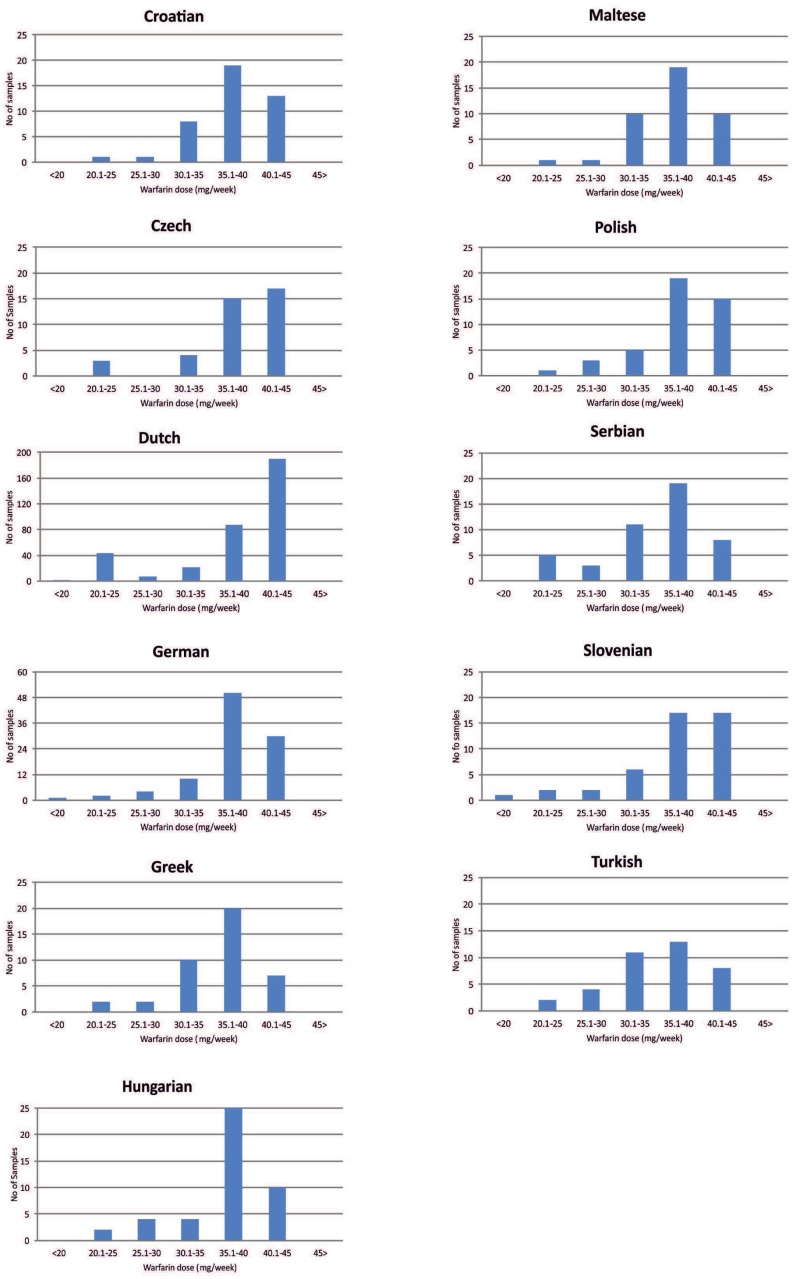
Distribution of the different individuals analyzed for each population group using the Affymetrix DMET^™^ Plus platform for the predicted weekly warfarin dose (mg).

We have then aimed to investigate the distribution of predicted warfarin dose among all individuals analyzed within the 11 population groups. Our data show that, with the exception of the Dutch and the Czech populations, the majority of individuals from all European populations analyzed should receive between 35.1–40 mg/week of the predicted warfarin dose (Fig 5). In the case of the Dutch population, the proportion of the individuals receiving the highest predicted warfarin doses (40.1–45 mg/week) is more than 2-fold higher compared to the individuals that should be receiving 35.1–40 mg/week, which explains the higher predicted warfarin doses for the Dutch population, compared to the European average (Fig 4). Overall, the vast majority of subjects from all 11 populations analyzed belong to the good warfarin metabolizers, deducted from the overall distribution of the predicted warfarin doses (Fig 5).

For the remaining populations, these overall differences in the weekly warfarin dose do not seem to be statistically significant, presumably due to the small sample size of these populations, but are nevertheless indicative of the dose requirements in each of them to achieve a therapeutic effect.

## Discussion

PGx holds promise for improving both the dosing and safety of existing drug treatment modalities and may assist in reducing geographic disparity in drug development. In this paper, we report our findings from a multicenter analysis of PGx biomarkers in a large number of European populations in an effort to reveal differences in the prevalence of several PGx biomarkers, particularly clinically actionable ones with direct impact on public health. We have also opted to study primarily samples from developing (low income) countries as a means to provide incentives to replicate this study in a much broader population sample which would, in turn, encourage PGx research and at the same time provide the basis for implementing PGx in routine clinical practice. As part of our genotyping effort, we have included samples from countries, such as Germany and the Netherlands, where PGx are implemented on a routine basis, e.g. in major healthcare centers. While one would anticipate the diversity for PGx biomarkers in various populations described herein, our data have important implications in (i) optimizing drug treatment at an individual level, by either increasing drug efficacy and/or reducing the risk for drug toxicity, and (ii) rationalizing healthcare costs expenditure at a national/population level. Also, we have implemented microattribution, an approach that provides credit and incentivizes data contributors towards data sharing, for putting together a research consortium and motivating PGx biomarker allele frequency data sharing. This process has been previously used to generate comprehensive genotype/phenotype information for *CFTR* gene variants from almost 40,000 patients [20]. We decided to implement microattribution in this project to encourage sample and data contribution, which enabled the construction of a very comprehensive repository of PGx biomarker allele frequency data at a European level, leading to over 35 research groups working together to either submit healthy volunteer DNA samples from their country, and/or PGx biomarker allele frequency data in return to direct microattribution credit.

Although in the past, there have been a number of studies to assess the prevalence of a small number of PGx biomarkers mostly in racial groups but also in distinct populations. Recently there have been some studies exploiting the Affymetrix DMET^™^ Plus platform to assess the prevalence of the same pharmacogenomic biomarkers in the Brazilian, Mexican [21] and Thai populations [22]. However, to our knowledge, our study has a number of very distinct features, namely (i) it addresses the prevalence of over 1,900 PGx biomarkers in 231 pharmacogenes, using one of the most comprehensive genetic screening platforms for PGx currently available, (ii) it comparatively analyzes a large number of, mostly Caucasian, populations, revealing a number of clinically actionable PGx biomarkers, whose prevalence differs significantly between these populations, which directly impact on the delivery of individualized treatment, (iii) apart from reporting the prevalence of a large number of PGx biomarkers, it also focuses on clinically actionable PGx biomarkers, which translates into the differential response of over 50 regulatory approved drugs. As such, our data confirm that ethnicity, even among closely related populations, indeed plays a significant role in differential drug response or toxicity and as such, it should be taken into consideration to reliably predict drug safety and efficacy at a population level. Alternatively, although population PGx analysis using indirect analysis tools, such as tagSNPs, imputed genotypes and/or ancestral markers [23,24] can be used, these approaches will never substitute the need to directly assess causative variants in clinical or public health policy decision making process. In the latter case, there are often subpopulation groups within a certain population with significant differences in the frequency of PGx biomarkers related to medication risk [25]. The latter may have implications in European populations, such as the Cypriot, the Hungarian, *etc*, that feature distinct subpopulation groups. Obviously, due to the pilot nature of the present study, we did not address this question in more detail, which could constitute the subject of larger and more population-focused projects. A recent study demonstrated that the Affymetrix DMET^™^ Plus platform can be effectively used to quantify population substructure [26].

Distinguishing population groups simply by performing PCA analysis and/or hierarchical clustering of Affymetrix DMET^™^ Plus platform genotyping data cannot lead to broad generalizations in the application of individual actionable variants, indicating that when clinical application of PGx information is needed, this is often driven by a very small number of clinically actionable variants. For example, although the Maltese, Turkish and the Greek populations display a similar profile on PCA analysis, yet these populations have significant differences in predicted warfarin dosing (Fig 4).

Assessment of the prevalence of clinically actionable pharmacogenotypes in this study further underlines the prospect of exploiting this knowledge in a clinical setting. The percentage of European subjects bearing actionable low- and high-risk pharmacogenotypes was relatively high (46.7% and 0.7%, respectively; Fig 3) and to those subjects, the homozygous normal individuals for these alleles should be also considered, since the latter genotypes are reassuring that the drug treatment will likely be efficacious. The results presented in this work have potentially a major public health impact, since they allow the formulation of medication prioritization guidelines. These guidelines could facilitate integration of PGx into the clinical practice, which can not only optimize the existing treatment modalities, but also contribute towards cost savings, which is of utmost importance for developing countries often facing huge fiscal deficits [27]. Indeed, we have observed significant differences in the genetic risk for either drug toxicity or efficacy with relevance for drugs used to treat high cholesterol levels, atrial fibrillation, and tuberculosis (Tables 2 and 3). Similarly, prior knowledge of the prevalence of the PGx biomarkers in different countries can help towards patient stratification for most populations during the drug development process. However, one should bear in mind that the frequency of a high-risk PGx biomarker alone is not adequate for developing national medication guidelines, but only in conjunction with other factors, such as disease burden.

Our study has a number of limitations. First of all, the number of samples in several populations is small, indicative of the pilot nature of this project. Nevertheless, we have made every effort to keep the sample size as proportionate to the size and representative of the structure of each population as possible so that our findings can help influence national care at a population level which has broader impact over a much larger number of patients. To this end, our findings indicate that extra caution should be exercised in the Polish population as far as simvastatin treatment is concerned, considering the statistical significance of the prevalence of the rs4149056 (SLCO1B1*5) PGx marker (Table 2), and the same seems to be true for clopidogrel and *CYP2C19* alleles and genotypes in the Greek population. Also, our data cannot be considered as representative for the entire European continent, since by focusing on populations from lower income European countries, we have excluded major European populations, such as the British, French and the Scandinavian in which PGx is already being implemented on a routine basis in the clinic. From a technical point of view, our genotyping strategy, based on the Affymetrix DMET^™^ Plus platform, does not take into account *CYP2D6* gene copy numbers or the CYP2D6*5 allele, which is an integral part of differences in *CYP2D6* expression and may lead to over-estimation of pharmacovariants allele frequencies.

## Conclusions

Herein, the microattribution approach was implemented towards the assessment of the pharmacogenomic biomarkers allelic spectrum in 18 European populations, mostly from developing European countries, by analyzing 1,931 pharmacogenomics biomarkers in 231 genes. Our findings indicate significant inter-population differences in pharmacogenomic biomarker allele frequency differences as well as the prevalence of high-risk genotypes. Such key findings became more profound when 7 clinically actionable pharmacogenomic biomarkers are considered in 7 European populations affecting drug efficacy and/or toxicity of 51 medication treatment modalities. Notable differences in predicted genotype-based warfarin dosing among these populations were also obtained.

## Future Perspectives

The key findings of this paper suggest that in the future the focus should be how national health authorities, stakeholders and policy makers involved in genomic medicine, particularly in developing European countries, to understand and be willing to use and possibly expand our findings to prioritize their medication choices or even amend their medicine policies. Such effort could give rise to the development of electronic tools and web-based applications to translate PGx information into a clinically meaningful format to assist physicians in their effort to individualize drug prescriptions on the basis of genomic information [28]. These efforts should be met with reciprocal activities to (i) enhance genomic literacy among physicians, such as cardiologists, oncologists, psychiatrists and neurologists who will stand at the frontline of genomic medicine and will be asked to exploit the new PGx knowledge to its full extent [29], (ii) precisely map the views and intentions of the key stakeholders and policy makers involved in implementing genomic medicine in an effort to expedite integration of pharmacogenomics into routine clinical care [30] and (iii) demonstrate that genome-guided drug treatment modalities are cost-effective in various developing countries to convince the policy makers to adopt this new discipline towards significant cost savings in their healthcare systems. Ultimately, such information would catalyze and possibly expedite the application of pre-emptive PGx testing with significant health benefits for the European citizens.

Lastly, one should bear in mind that the genotyping approach described above, though comprehensive, cannot possibly substitute the application of whole genome sequencing in pharmacogenomics [31], not only for the total number of genomic biomarkers to be identified in the pharmacogenes, but most importantly for the identification of rare novel and putatively deleterious variants which may render their carriers intermediate or even poor metabolizers to certain medications. Nevertheless, an alternative approach, which can be proved particularly useful for developing countries, would be the establishment of specific genotyping panels, e.g. population-specific or European, that could be used preemptively prior to or at the point of taking the required medication, hence offering the added value of timeliness. Previous work has shown that almost 2/3 of patients in the clinic receive a drug with pharmacogenomic information in their labels [32], which demonstrates that such an approach would not only be considered as an attractive alternative, which can also likely be cost-effective, reducing healthcare expenditure.

## Supporting Information

S1 File **Table A**. Minor allele frequencies of the 36 main actionable pharmacogenomic biomarkers that have been analyzed among the different population groups [number of samples (n) analyzed are shown underneath]. Minor allele frequencies in boldface are statistically significant compared to the European average (please refer to Table 2 for the corresponding p-values). **Table B**. Prevalence of the PGx biomarkers, based on the star alleles nomenclature of individuals in the populations analyzed by the Affymetrix DMET^™^ Plus platform. *Abbreviations (as defined by the DMET console)*: UNIQ+UNK: Unique annotated haplotype pair, with other haplotype pairs requiring unannotated haplotypes also possible; MULT+UNK: Multiple annotated haplotype calls possible, with other haplotype pairs requiring unannotated haplotypes also possible; NC/PRA/NA: NoCall, PossibleRareAllele, or NotAvailable call for one or more markers resulting in multiple haplotype pairs. **Table C**. Summary of the various commercially available TaqMan Assay IDs for each of the actionable pharmacogenomic biomarkers screened in this study.
(XLSX)

S2 File Figure A. Ancestry analysis of 11 European populations analyzed using the Affymetrix DMET^™^ Plus platform. Y-axis indicated the ancestry percentage for every individual analyzed using the Affymetrix DMET^™^ Plus platform (displayed in the x-axis). Colors represent the percentage of the different ancestries in each individual (Europeans in green, Asians in red and Africans in blue).
(DOCX)
