# Correction: A European Spectrum of Pharmacogenomic Biomarkers: Implications for Clinical Pharmacogenomics

**DOI:** 10.1371/journal.pone.0172595

**Published:** 2017-02-16

**Authors:** Clint Mizzi, Eleni Dalabira, Judit Kumuthini, Nduna Dzimiri, Istvan Balogh, Nazli Başak, Ruwen Böhm, Joseph Borg, Paola Borgiani, Nada Bozina, Henrike Bruckmueller, Beata Burzynska, Angel Carracedo, Ingolf Cascorbi, Constantinos Deltas, Vita Dolzan, Anthony Fenech, Godfrey Grech, Vytautas Kasiulevicius, Ľudevít Kádaši, Vaidutis Kučinskas, Elza Khusnutdinova, Yiannis L. Loukas, Milan Macek, Halyna Makukh, Ron Mathijssen, Konstantinos Mitropoulos, Christina Mitropoulou, Giuseppe Novelli, Ioanna Papantoni, Sonja Pavlovic, Giuseppe Saglio, Jadranka Sertić, Maja Stojiljkovic, Andrew P. Stubbs, Alessio Squassina, Maria Torres, Marek Turnovec, Ron H. van Schaik, Konstantinos Voskarides, Salma M. Wakil, Anneke Werk, Maria del Zompo, Branka Zukic, Theodora Katsila, Ming Ta Michael Lee, Alison Motsinger-Rief, Howard L. Mc Leod, Peter J. van der Spek, George P. Patrinos

The thirty-third author's name is spelled incorrectly. The correct name is: Jadranka Sertić

## References

[1] Mizzi C, Dalabira E, Kumuthini J, Dzimiri N, Balogh I, Başak N, et al (2016) A European Spectrum of Pharmacogenomic Biomarkers: Implications for Clinical Pharmacogenomics. PLoS ONE 11(9): e0162866 doi: 10.1371/journal.pone.0162866 27636550 10.1371/journal.pone.0162866 PMC5026342

