# Supplementary material for: A European Spectrum of Pharmacogenomic Biomarkers: Implications for Clinical Pharmacogenomics
Source: PLoS One. 2016 Sep 16;11(9):e0162866. doi: 10.1371/journal.pone.0162866 (PMC5026342; doi:10.1371/journal.pone.0162866)
Supplement: S2 File — Ancestry analysis of 11 European populations analyzed using the Affymetrix DMET™ Plus platform. Y-axis indicated the ancestry percentage for every individual analyzed using the Affymetrix DMET™ Plus platform (displayed in the x-axis). Colors represent the percentage of the different ancestries in each individual (Europeans in green, Asians in red and Africans in blue). (DOCX) [file pone.0162866.s002.docx]

**S2 file**

**A European spectrum of pharmacogenomic biomarkers: Implications for clinical pharmacogenomics**

Clint Mizzi ^1,2^, Eleni Dalabira ^3,#^, Judit Kumuthini ^4^, Nduna Dzimiri ^5^, Istvan Balogh ^6^, Nazli Başak ^7^, Ruwen Böhm ^8^, Joseph Borg ^9^, Paola Borgiani ^10^, Nada Bozina ^11^, Henrike Bruckmueller ^8^, Beata Burzynska ^12^, Angel Carracedo ^13^, Ingolf Cascorbi ^8^, Constantinos Deltas ^14^, Vita Dolzan ^15^, Anthony Fenech ^16^, Godfrey Grech ^16^, Vytautas Kasiulevicius ^17^, Ľudevít Kádaši ^18,19^, Vaidutis Kučinskas ^17^, Elza Khusnutdinova ^20,21^, Yiannis L. Loukas ^22^, Milan Macek Jr ^23^, Halyna Makukh ^24^, Ron Mathijssen ^25^, Konstantinos Mitropoulos ^26^, Christina Mitropoulou ^25^, Giuseppe Novelli ^10^, Ioanna Papantoni ^3^, Sonja Pavlovic ^27^, Giuseppe Saglio ^28^, Jadranka Setric ^11,29^, Maja Stojiljkovic ^27^, Andrew P. Stubbs ^1^, Alessio Squassina ^30^, Maria Torres ^13^, Marek Turnovec ^23^, Ron H. van Schaik ^25^, Konstantinos Voskarides ^14^, Salma M Wakil ^5^, Anneke Werk ^8^, Maria del Zompo ^30^, Branka Zukic ^27^, Theodora Katsila ^3^, Ming Ta Michael Lee ^31,##^, Alison Motsinger-Rief ^32^, Howard L. Mc Leod ^33^, Peter J. van der Spek ^1^, George P. Patrinos ^1,3,^*

^1^ Erasmus University Medical Center, Faculty of Medicine, Department of Bioinformatics, Rotterdam, the Netherlands; ^2^ University of Malta, Faculty of Medicine and Surgery, Department of Physiology and Biochemistry, Msida, Malta; ^3^ University of Patras School of Health Sciences, Department of Pharmacy, Patras, Greece; ^4^ Center for Proteomic and Genomic Research, Observatory, Cape Town, South Africa; ^5^ King Faisal Specialist Hospital and Research Centre, Riyadh, Saudi Arabia; ^6^ University of Debrecen, Debrecen, Hungary; ^7^ Boğaziçi University, Istanbul, Turkey; ^8^ University of Kiel, Institute for Experimental and Clinical Pharmacology, Kiel, Germany; ^9^ University of Malta, Department of Applied Biomedical Science, Faculty of Health Sciences, Msida, Malta; ^10^ University of Rome “Tor Vergata”, Department of Biomedicine and Prevention, Rome, Italy; ^11^ University Hospital Centre, Zagreb, Croatia; ^12^ Institute of Biochemistry and Biophysics, Polish Academy of Sciences, Warsaw, Poland; ^13^ University of Santiago de Compostela, Santiago, Spain; ^14^ University of Cyprus, Molecular Medicine Research Center, Department of Biological Sciences, Nicosia, Cyprus; ^15^ University of Ljubljana Faculty of Medicine, Ljubljana, Slovenia; ^16^ University of Malta, Faculty of Medicine, Department of Surgery, Msida, Malta; ^17^ Department of Human and Medical Genetics, Faculty of Medicine, Vilnius University, Vilnius, Lithuania; ^18^ Comenius University, Faculty of Natural Sciences, Bratislava, Slovakia; ^19^ Center for Molecular Medicine, Slovak Academy of Sciences, Bratislava, Slovakia; ^20^ Institute of Biochemistry and Genetics, Ufa Scientific Center, Russian Academy of Sciences, Ufa, Russia; ^21^ Department of Genetics and Fundamental Medicine, Bashkir State University, Ufa, Russia; ^22^ University of Athens, Faculty of Pharmacy, Department of Pharmaceutical Chemistry, Athens, Greece; ^23^ Charles University, 2^nd^ Faculty of Medicine and University Hospital Motol, Prague, Czech Republic; ^24^ Institute of Hereditary Pathology, Ukrainian National Academy of Medical Sciences, Lviv, Ukraine; ^25^ Erasmus University Medical Center, Department of Clinical Chemistry, Rotterdam, the Netherlands; ^26^ The Golden Helix Foundation, London, UK; ^27^ Institute of Molecular Genetics and Genetic Engineering University of Belgrade, Laboratory of Molecular Biomedicine, Belgrade, Serbia; ^28^ University of Turin School of Medicine, Turin, Italy; ^29^ University of Zagreb School of Medicine, Zagreb, Croatia; ^30^ University of Cagliari, Department of Biomedical Sciences, Cagliari, Italy; ^31^ RIKEN Institute, Center for Genomic Medicine, Laboratory for International Alliance, Yokohama, Japan; ^32^ North Carolina State University, Department of Statistics, Raleigh, NC, USA; ^33^ Moffitt Cancer Center, Tampa, FL, USA

Present addresses:

^#^: Lausanne University Hospital, Lausanne, Switzerland

^##^: Geisinger Genomic Medicine Institute, Danville, PA, USA

**Figure A in S2 File**

**
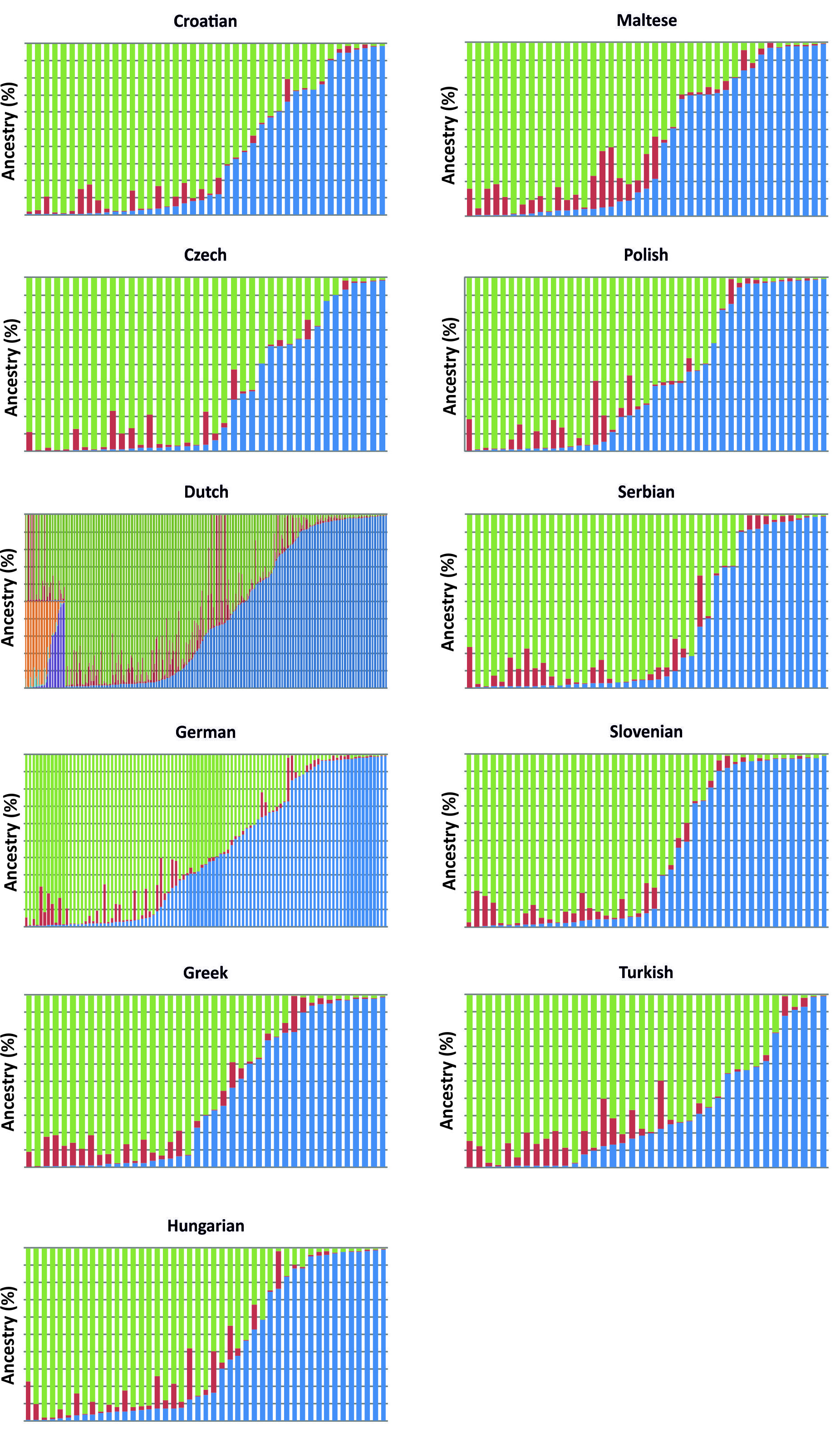
**
